# Clinical data analysis of telmisartan for hypertension management in Indian population

**DOI:** 10.6026/97320630017652

**Published:** 2021-06-30

**Authors:** A Prem Kumar, Anirudra Ghorai, Vasudev Kriplani, Rabindra Kumar Dash, J Aravinda, Paramesh Shamanna, TK Sabeer, Abdul Hannan, Mahesh Abhyankar, Santosh Revankar

**Affiliations:** 1 DiaPlus Clinic, Krishnammal Nagar, Fairlands, Salem, Tamilnadu- 636016, India; 2 Divine nursing home, Taljuli Dr. Dandapat Complex, Kharagpur, West Bengal - 721301, India; 3 Kriplani nursing Home, E-Ward, Tarabai Park, Kolhapur, Maharashtra - 416003,India; 4 Gupta Diagnostic and Research Centre, Deulasahi, Bhanjpur, Baripada, Odisha - 757001, India; 5 Dr.Aravind's Diabetes Center,No. 14 & 15, 7th Main, 3rd Block, 4th Stage, BasaveshwarNagar, Bengaluru, Karnataka-560079, India; 6 Bangalore Diabetes Centre, No.426, 4th Cross Rd, HBR Layout 2nd Block, Stage 1, Kalyan Nagar, Bengaluru, Karnataka 560043, India; 7 Diacare, 2nd Floor, Chamber Plaza, Thayatheru Rd, Thayatheru, Thana, Kannur, Kerala-670002, India; 8 Dr. Hamdulay's Cardiac Rehabilitation Centre, 233/234,Bellasis Road, Junction, Nagpada, Mumbai - 400008, India; 9 USV Private Limited, BSD Marg, Station Road, Deonar, Govandi East, Mumbai, Maharashtra - 400088, India

**Keywords:** Hypertension, Telmisartan, Blood pressure, therapeutic compliance, combinatorial therapy

## Abstract

It is of interest to evaluate the clinical characteristics, treatment patterns, clinical effectiveness, and safety of telmisartan as a monotherapy or as part of combination therapy in Indian adults (>18 years old) with hypertension. All patients were receiving telmisartan as monotherapy, or as a combination therapy for hypertension management. Demographics, risk factors, existing comorbidity, and ongoing medical therapies were retrieved from the patients’ medical records. A total of 8607 patients with hypertension (median age, 51.0 years) were part of the study. The gender distribution suggested, 5534(64.3%) patients were male, and 3073 (35.7%) were female patients. The excess salt intake (39.0%) was the most common risk factor according to the results. The analysis revealed telmisartan dual therapy (57.9%) as the most prescribed therapy, followed by monotherapy (32.5%), and triple therapy (9.6%). Further, telmisartan 40mg (21.3%) and telmisartan 40mg plus amlodipine 5mg (17.6%) were the most commonly prescribed therapies. The data suggested that only 17.2% of patients required dose titration. The mean systolic blood pressure (SBP) and diastolic blood pressure (DBP) (mmHg) were significantly decreased with monotherapy (mean change: 19.8 [15.1] mmHg and 8.8[8.2] mmHg), dual therapy (mean change: 23.7 [16.6] mmHg and 10.3[8.5] mmHg), and triple therapy (mean change: 28.6 [19.0] mmHg and 12.1[10.8] mmHg) after the treatment (P<0.001). A total of 98.4% of the patients were compliant, and 97.6% achieved the target blood pressure goal with telmisartan-based therapy. There were 157 adverse events reported altogether. The Physicians' global evaluation of efficacy and tolerability showed the majority of the patients receiving telmisartan-based therapy on a good to excellent scale. Telmisartan used as a monotherapeutic agent or as a part of combination therapy was successful and effective in reducing blood pressure and achieving the blood pressure target. Irrespective of the patient’s age, duration, and stages of hypertension, the study resulted in a good to excellent scale in efficacy and tolerability in the Indian patients having hypertension.

## Background

Hypertension is one of the leading causes of the increasing global deaths due to cardiovascular diseases (CVDs) and chronic kidney diseases (CKDs) [1] 230 million adults are suffering from hypertension in India [2]. Study reports suggest that more than half of hypertension patients have uncontrolled blood pressure (BP) in India [3-4]. An increased prevalence of high blood pressure in young Indian adults has become a serious health concern [2-5]. Indian patients should be educated about the benefits of lifestyle modification, treatment, and compliances,which may help in achieving the targeted blood pressure control in the population [6]. Dual-drug combination treatment initiation,preferably in a single pill for stage II hypertension is also recommended [6-8]. ARBs as anti-hypertensive agents are the most common component of dual and triple therapies in India [2,9,10]. Most physicians prefer telmisartan, an ARB, because of its continual effectiveness, morning BP surge control, and prevention of microalbuminuria, nephropathy, cardiovascular morbidity, and mortality [2]. Therefore, it is of critical importance to conduct clinical data analysis of telmisartan for hypertension management in the Indian population.

## Methods

### Study design and ethical approval

This study was a retrospective, multicentre, observational, and real-world study conducted at 331 sites across Indian healthcare centers. Patients having medical records with diagnosed hypertension, and who were receiving telmisartan as monotherapy and/or combination therapy for hypertension were included. The study was approved by the Independent Ethics Committee (IEC), Clinicom, Bangalore. The study procedure was in accordance with the principles of the Declaration of Helsinki,the International Conference on Harmonization Good Clinical Practices (ICH GCPs), and the applicable legislation on noninterventional studies.

### Study population

Patients of either sex, aged above 18 years, diagnosed with hypertension as per the American College of Cardiology (ACC) or American Heart Association (AHA) criteria (ACC/AHA guidelines 2017), and receiving treatment for hypertension with telmisartan monotherapy and/or combination therapy, were included in this study. According to the ACC/AHA criteria, normal BP is defined as <120/<80 mmHg, elevated BP as 120-129/<80 mmHg, hypertension stage 1 as 130-139/80-89 mmHg,and hypertension stage 2 as ≥140/≥90 mmHg [11]. Investigators' discretion and the decision were considered for excluding the patients having incomplete data or any specific unsuitable conditions.

### Data collection

The data was collected from the existing case record forms that included demographic data, lifestyle associated information,family history, treatment history, and therapy details. The demographic data was having information on age, gender,height, and weight. The lifestyle-related information included physical activity, smoking history, and alcohol consumption. The family history of hypertension, dyslipidemia, diabetes mellitus,stage or grade of hypertension, and duration of hypertension was recorded. The hypertension treatment history and current telmisartan therapy, dosage, and duration of telmisartan as monotherapy and/or combination therapy were also accounted for. Other crucial information, such as the current status of hypertension (controlled/uncontrolled) and any adverse events related to telmisartan, were included in the study.

### Statistical analysis:

Data were analyzed using Statistical Package for The Social Sciences (SPSS) software (version 23.0). Demographic characteristics included median and interquartile range (IQR) for the continuous variables and frequency and percentages for the categorical variables. A comparison of qualitative variables between the groups was done using the chi-square test, and the Mann-Whitney U test was used for the quantitative variables. A paired sample t-test was used for comparing the pre-and posttreatment systolic BP (SBP) and diastolic BP (DBP). A P-value less than 0.05 were considered statistically significant.

## Results:

### Patient distribution:

A total of 8607 patients with hypertension were enrolled. The median age of the patients was 51.0 years. The number of male patients (64.3%) was higher than the number of female patients (35.7%). The majority of the patients (51.7%) were from urban locations. A total of 64.4% of the patients were diagnosed with stage II hypertension, and the remaining patients (35.6%) having stage I hypertension. The median systolic blood pressure (SBP) and diastolic blood pressure (DBP) were 155.0 mmHg and 94.0 mmHg, respectively (Table 1 - see PDF).

### Risk factors assessment:

**Figure 1:**
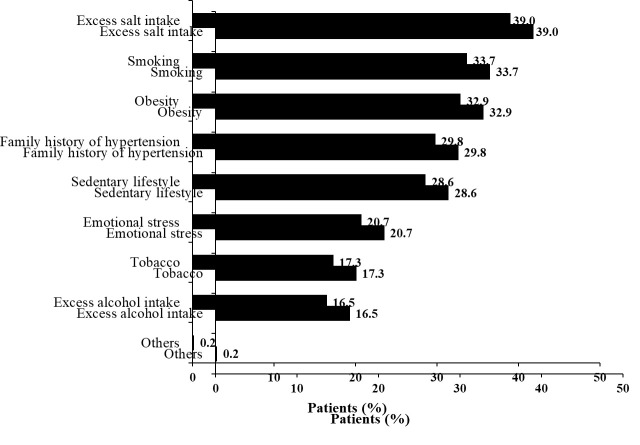
Risk factors in clinical study are shown; *Other factors include chronic kidney disease, diabetes mellitus, dyslipidemia,thyroid

It was observed that excess salt intake (39.0%) was the most common risk factor among the overall population. Other important risk factors were smoking (33.7%), obesity (32.9%),family history of hypertension (29.8%), sedentary lifestyle (28.6%), emotional stress (20.7%), tobacco consumption (17.3%) and excess alcohol intake (16.5%) (Figure 1).

### Therapeutic evaluation of Telmisartan:

The monotherapy and combination therapy of telmisartan was received by 32.5% and 67.5% of the patients, respectively. In combination therapy, dual therapy was the most commonly prescribed therapy (85.8%), whereas triple therapy was prescribed for 14.2% of the patients only. The 65.5% of patients having monotherapy were prescribed telmisartan 40 mg dose. Other patients undergoing monotherapy, were on telmisartan 80 mg (20.1%) and telmisartan 20 mg (14.4%). In combination therapy, the majority of the patients were prescribed telmisartan and amlodipine (39.1%). This was followed by other combinations such as telmisartan and chlorthalidone (25%),telmisartan and hydrochlorothiazide (18.7%), and telmisartan,and metoprolol succinate (17.2%). The most common treatment regime of the dual combination therapy was telmisartan 40 mg and amlodipine 5 mg dose (30.5%). Triple combination therapy was prescribed for 823 patients. The most commonly prescribed triple-drug combination therapy was telmisartan 40 mg,amlodipine 5 mg, and hydrochlorothiazide 12.5 mg (79.3%) (Table 2 - see PDF).

### Treatment duration, dose titration, and prior therapy:

The median duration of the treatment was 12.0 months. The dose titration was done only for 1479 patients (17.2%). The majority of the patients (81.3%) had dosage up-titration and 18.1% of the patients had dosage down-titration during the treatment. Before the telmisartan-based therapy, a total of 22.4% of the patients were treated with other antihypertensive.

### Telmisartan therapy outcome:

**Figure 2:**
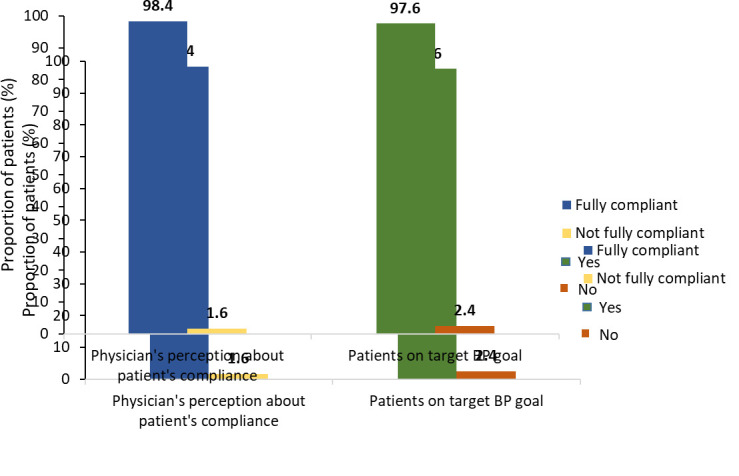
Physician perception of patient compliance with fixeddose combination therapy and proportion of patient on target BP goal with telmisartan-based therapy. Fully compliant: greater than 80% of prescribed medication; Not fully compliant: less than 80% prescribed medication

**Figure 3:**
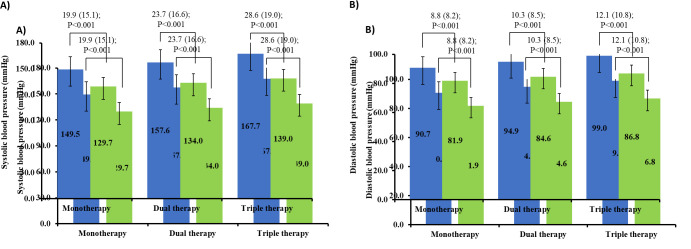
Mean (SD) change in A) SBP and B) DBP level from pre to post-treatment. Data shown as mean change (SD); P-value

Analysis of the patient compliance suggested that a total of 98.4% of patients were compliant, and 97.6% of patients achieved the target BP goal with telmisartan-based therapy (Figure 2). On the other hand, a total of 157 patients reported adverse events. The results further suggest that the mean SBP significantly decreased after the monotherapy, dual therapy, and triple therapy of telmisartan. The mean (SD) change of 19.9(15.1) mm Hg (P<0.001) observed for the monotherapy, 23.7(16.6) mm Hg (P<0.001) for dual therapy, and 28.6(19.0) mm Hg (P<0.001) for triple therapy. Similarly, the mean DBP was also found to significantly decreased post-treatment evaluations. The DBP mean (SD) change of 8.8 (8.2) mm Hg (P<0.001) was observed for monotherapy, 10.3(8.5) mm Hg (P<0.001) for dual therapy, and 12.1(10.8) mm Hg (P<0.001) for the triple therapy (Figure 3). Further analysis revealed that the median SBP and DBP increased significantly with the growing age (P<0.001). In the elderly patient population (>60 years), stage II hypertension was common. An abundance of Stage I hypertension was observed in the young and adult (<18-≤45 years) patient group (P<0.001). Significant dosage up-titration was recorded compared to the dosage down-titration in the patients receiving mono, dual or triple therapy (P<0.001). Physician's global evaluation of efficacy and tolerability showed the majority of the patients receiving either monotherapy (98.5% and 91.4%), dual therapy (98.7% and 95.1%), or triple therapy (98.3% and 97.3%), reported having a good to excellent scale evaluation (Table 4 - see PDF).

## Discussion:

Hypertension is a growing serious health problem in India causing a significant burden on the existing health care system. Indians are prone to hypertension and related complications due to the early onset of hypertension, multiple CVD risk factors, lifestyle problems, lack of awareness on health, treatment, and BP control [6]. Telmisartan supports a long duration of blood pressure control, possesses high lipophilicity that enhances tissue penetration, intracellular absorption, and bioavailability, and may provide vascular protection. Telmisartan is shown to provide optimal cardioprotection along with a good tolerance profile [12]. This real-world study documented the clinical characteristics, and treatment patterns of telmisartan. This study included dosage types and the use of telmisartan as an important drug for monotherapy and combination therapy in adult patients having hypertension. The patients were considered from 331 clinical study centers across India. Moreover, this study also evaluated the clinical effectiveness and safety of telmisartan use for monotherapy and combination therapy for hypertensive patients. The most commonly used dosage in monotherapy was telmisartan 40 mg, and in the dual therapy, it was telmisartan 40 mg and amlodipine 5 mg. Many reports suggested the combination of telmisartan 40 mg and amlodipine 5mg as efficacious, especially for patients who failed to respond adequately to monotherapy. In patients with uncontrolled hypertension receiving monotherapy of amlodipine 5 mg, the fixed-dose combination of telmisartan 40 mg and amlodipine 5 mg was effective. These dose combinations significantly reduced themean BP assessed for 24-hour, however, the administration time of the drug combination did not influence the BP reduction outcome [13]. Similarly, telmisartan 40 mg has been widely effective in patients with mild to moderate hypertension [14]. Other studies conducted on Indian hypertension patients demonstrated that telmisartan 40mg significantly reduced the SBP and DBP along with favorable effects on blood glucose, lipids, and heart rate [15,16]. The selection of mono or combination therapy was done based on the individual demographic, anthropometric characteristics, concomitant cardiovascular risk factors, asymptomatic organ damage, BP target, and other clinical conditions [17]. Often, antihypertensive drugs may require dose titration to achieve the desired BPlowering effect while maintaining tolerability. Failure of specific antihypertensive medication dose in achieving desired BPlowering effect may require up-titration of the dose to improve BP control [18]. In the current study, very few patients required dosage titration compared to their respective initial telmisartanbased therapeutic dose. In this study, about 22.4% of the patients were treated with other antihypertensive drugs before the telmisartan-based therapy. No prior antihypertensive drugs were used for 77.6% of the patients. Reports suggest that irrespective of the earlier treatment status, telmisartan-based therapies were efficacious for BP reduction in hypertension patients [19,20]. In this study, 98.4% of the patients were compliant with the telmisartan-based regimen demonstrating the efficacy of telmisartan as a mono therapeutic agent or as a part of combination therapy in controlling hypertension. The results were in accordance with the reported study suggesting that >97% of the study population attained the targeted BP using mono and combination therapy of this drug. Further, the compliance rate was also found consistent with another report [21]. The tolerability of telmisartan was reported acceptable in the earlier global and Indian studies [18-22]. The present study also has acceptable tolerability, and only 1.8% of the patients experienced some minor adverse events during the monotherapy and combination therapy. The applied physicians' global evaluation of efficacy and tolerability suggested the majority of the patients reported the results within the good to excellent scale. Evaluation of the SBP and DBP reduction suggested, that the better BP reduction was achieved using the triple therapy, followed by dual and monotherapy of telmisartan. Overall, all types of therapies adopted using the telmisartan were efficacious. An earlier report suggested that the combination therapy demonstrated better outcomes for achieving optimal BP control in the study population [18]. Another report conducted as a prospective, open-label, non-comparative, post-marketing surveillance analysis, suggested the dual combination therapy of telmisartan and hydrochlorothiazide/amlodipine was effective in SBP and DBP reduction significantly for Indian hypertension patients [23].

We have noted that the key risk factors for hypertension were excess salt intake, smoking, obesity, family history of hypertension, sedentary lifestyle, emotional stress, tobacco chewing, and excess alcohol intake. These findings were in agreement with the previous Indian studies. According to the National Family Health Survey (NFHS) 2015-16, increasing age, obesity, overweight, male gender, urban lifestyle, and alcohol consumption were the crucial independent risk factors for Indian adults with hypertension [24]. Similarly, increasing age, parental history of hypertension, tobacco use, physical inactivity, high estimated per capita salt consumption, and BMI ≥27.5 kg/m2, were found as the risk factors of hypertension in a communitybased, cross-sectional study conducted in central India [25]. In patients having hypertension and dyslipidemia, the application of fixed-dose combinations using telmisartan, and other drugs such as rosuvastatin and amlodipine, was successful in controlling the BP and LDL-C level. This fixed-dose therapeutic combination was safe and tolerable [26]. A clinical study conducted by Coleman et al. [27] suggested that telmisartan was effective in controlling the SBP. An open-label clinical trial was conducted for the effectiveness of azilsartan and telmisartan on patients having diabetes and hypertension. Both the drugs displayed antihypertensive effects successfully, however, no clinically significant insulin resistance effect was observed for azilsartan and telmisartan [28]. Verdecchia reported that telmisartan is effective in reversing cardiac remodelling through improving the left ventricular and left atrial functions in hypertensive patients [29]. Alongside cardiac health improvement, telmisartan was reported to have an impact on the growth of small abdominal aortic aneurysms [30]. Other clinical efficacy and safety studies also recommended telmisartan as an effective therapeutic agent in managing blood pressure, cardiovascular risks, and renovascular conditions [31-32]. The superiority of telmisartan in comparison to other similar drugs in controlling BP has been reported earlier along with better cardiovascular organ protection [33-34]. Clinical pharmacological analysis suggested that telmisartan is an angiotensin II type-1 (AT1) receptor blocker that has a comparatively higher affinity towards AT1 rather than AT2 [35]. Telmisartan did not show any rebound phenomenon and tolerance and also displayed a prolonged elimination half-life [36]. Similar to the present study, management of hypertension in patients having type 2 diabetes using telmisartan and other drug combination was reported earlier [37]. The results obtained in the present analysis suggest that telmisartan is an effective and safe medication, used alone or in combination with other drugs for controlling BP in hypertensive patients or patients having type 2 diabetes.

## Limitations:

Several parameters such as the antihypertensive regimen used beforetelmisartan-based therapy; details of the concomitant medication, and time of previous visits could not be captured. Such information will have an indirect effect on the overall study results. Further, a large-scale, prospective, well-designed analysis will help to establish the efficacy and safety of telmisartan-based regimens in the Indian population.

## Conclusion

Analysis of 8607 hypertension patients suggested that telmisartan is efficacious and tolerable for BP control when used as part of monotherapy and in combination therapy for Indian patients. This is effective irrespective of age, duration, and stages of hypertension; the therapies were tolerable by the study population with few minor adverse events.
